# The decision for or against mycoparasitic attack by *Trichoderma* spp. is taken already at a distance in a prey-specific manner and benefits plant-beneficial interactions

**DOI:** 10.1186/s40694-024-00183-4

**Published:** 2024-09-09

**Authors:** Pia Stange, Johannes Kersting, Prasath Balaji Sivaprakasam Padmanaban, Jörg-Peter Schnitzler, Maaria Rosenkranz, Tanja Karl, J. Philipp Benz

**Affiliations:** 1 Professorship for Fungal Biotechnology in Wood Science, Wood Research Munich, TUM School of Life Sciences, Technical University of Munich, Freising, Germany; 2 Chair of Experimental Bioinformatics, TUM School of Life Sciences, Technical University of Munich, Freising, Germany; 3 Research Unit Environmental Simulation, Helmholtz Munich, Neuherberg, Germany; 4 Present Address: Institute of Plant Sciences, Ecology and Conservation Biology, University of Regensburg, Regensburg, Germany

**Keywords:** Antibiosis, Biocontrol agent, Chemotropism assay, Ectomycorrhizal, Host sensing, Mycoparasitism, Plant-pathogenic fungi, *Trichoderma* spp.

## Abstract

**Background:**

The application of plant-beneficial microorganisms as bio-fertilizer and biocontrol agents has gained traction in recent years, as both agriculture and forestry are facing the challenges of poor soils and climate change. *Trichoderma* spp. are gaining popularity in agriculture and forestry due to their multifaceted roles in promoting plant growth through e.g. nutrient translocation, hormone production, induction of plant systemic resistance, but also direct antagonism of other fungi. However, the mycotrophic nature of the genus bears the risk of possible interference with other native plant-beneficial fungi, such as ectomycorrhiza, in the rhizosphere. Such interference could yield unpredictable consequences for the host plants of these ecosystems. So far, it remains unclear, whether *Trichoderma* is able to differentiate between plant-beneficial and plant-pathogenic fungi during the process of plant colonization.

**Results:**

We investigated whether *Trichoderma* spp. can differentiate between beneficial ectomycorrhizal fungi (represented by *Laccaria bicolor* and *Hebeloma cylindrosporum*) and pathogenic fungi (represented by *Fusarium graminearum* and *Alternaria alternata*) in different confrontation scenarios, including a newly developed olfactometer “race tube”-like system. Using two independent species, *T. harzianum* and *T. atrobrunneum*, with plant-growth-promoting and immune-stimulating properties towards *Populus* x *canescens*, our study revealed robustly accelerated growth towards phytopathogens, while showing a contrary response to ectomycorrhizal fungi. Transcriptomic analyses identified distinct genetic programs during interaction corresponding to the lifestyles, emphasizing the expression of mycoparasitism-related genes only in the presence of phytopathogens.

**Conclusion:**

The findings reveal a critical mode of fungal community interactions belowground and suggest that *Trichoderma* spp. can distinguish between fungal partners of different lifestyles already at a distance. This sheds light on the entangled interactions of fungi in the rhizosphere and emphasizes the potential benefits of using *Trichoderma* spp. as a biocontrol agent and bio-fertilizer in tree plantations.

**Supplementary Information:**

The online version contains supplementary material available at 10.1186/s40694-024-00183-4.

## Background

Filamentous fungi of the genus *Trichoderma* are naturally occurring in soil and on plant surfaces [1, 88]. The genus has been widely studied as biocontrol agents (BCA) [10, 40, 112, 116, 119]. Biocontrol is based on several mechanisms, such as antagonistic activity against fungal plant pathogens, proficiency to colonize plant tissues, the ability to induce systemic resistance in plants, as well as the adaptability to a wide range of environments [65, 73, 105]. During the complex process of mycoparasitism, coherent mechanisms of antibiosis, competition for nutrients and space and direct inhibition by the release of fungal cell wall degrading enzymes, such as chitinases, proteases, and β-glucanases, can effectively lead to inhibition and death of prey fungi [73, 113, 123, 128]. The application of *Trichoderma* as BCA in disease management has already been shown to be effective against a broad range of foliar and root pathogens [2, 8, 125].

In addition to functioning as bio-fungicide, numerous rhizosphere-competent *Trichoderma* spp. can form close symbioses with plants, producing soluble metabolites and volatile organic compounds (VOCs) with plant-performance stimulating activities conferring improved growth and induced resistance to abiotic and biotic stresses [34, 48, 85, 126]. Therefore, the application of *Trichoderma* spp. can minimize the amount of traditional fertilizers by improving nutrient and water acquisition, as well as reducing the amount of synthetic fungicides [52, 56, 66, 114].

Hybrid poplars (*Populus* spp.) have gained significance due to their fast growth, making them valuable feedstocks for a range of wood and non-wood products with high economic importance [94, 96, 103]. *Populus* spp. are naturally found in symbiotic interactions with ectomycorrhizal fungi (ECM) [58, 60, 69, 93, 109]. However, monocultures of poplar hybrids in large-scale short rotation coppices (SRC) are also often susceptible to a wide range of soil-born and foliar fungal pathogens such as *Armillaria* root rot, *Melampsora* leaf rust, or leaf spot caused by *Alternaria alternata* [42, 89]*.* Despite *A. alternata* being recognized as an airborn pathogen, its chlamydospores have been observed to persist both in soil and infested organic matter and was found on diseased roots of *Vaccinium corymbosum* and *Taxus* x *media* [84]. *Trichoderma* spp. isolated from the rhizosphere have been shown to display a high antagonistic activity against common poplar pathogens, including *A.* *alternata* [4, 137]. Another economically important phytopathogen is the ascomycete *Fusarium graminearum*, infecting a wide range of cereal crops such as e.g. wheat, barley, or maize [46]. The most common disease caused by *F. graminearum* is Fusarium head blight (FHB) [17, 22]. Several studies have suggested *Trichoderma* spp. to act as effective biocontrol agents against this pathogen [63, 75, 110]. However, the mycotrophic nature of *Trichoderma* bears the potential to negatively affect the local ECM population through competition for essential nutrients, growth inhibition, or direct antagonism [31, 33, 120]. While *Trichoderma*-based BCA might thus be a promising tool in SRCs to mitigate susceptibility to phytopathogenic fungi and improve biomass productivity, further research is needed to study the interactions between *Trichoderma* and plant-beneficial fungi in the rhizosphere, such as ECM, to evaluate potential risks of adverse effects on these non-target associations [78], as these interactions play crucial roles in soil health and nutrient uptake [32, 70, 76].

Existing literature reflects a degree of ambiguity regarding the ability of *Trichoderma* to differentiate between diverse fungal taxa, with important implications on the framework of the plant holobiont theory [51, 141]. Understanding whether *Trichoderma* can selectively interact with certain fungi while resisting others would provide invaluable insights into the complex dynamics of plant-fungus interactions and ecosystem functioning. We therefore initiated a study to directly investigate the ability of *Trichoderma* spp. to distinguish between plant-beneficial ECM and plant-pathogenic fungi using representative species of each of these two major lifestyle groups. For this purpose, we selected two *Trichoderma* wild-type strains from the Harzianum clade (one *T. harzianum* and one *T. atrobrunneum* strain). Confrontation scenarios were set up with two representative phytopathogens: *A. alternata* and *Fusarium graminearum*, both causing devastating diseases and mycotoxin contaminations worldwide [135]. The interaction with the phytopathogens was investigated in direct comparison with *Laccaria bicolor* and *Hebeloma cylindrosporum* as representative ECM. To evaluate the physiological response of *Trichoderma* over longer distances and time while having a two-directional choice of growth, a novel olfactometer “race tube”-like system was developed. Moreover, transcriptomics was used to detect differences in the initiation of mycoparasitism-related programs along the contact stages and to investigate the interaction on a molecular level.

## Methods

### Cultures conditions

The *Trichoderma* strain *T. harzianum* WM24a1 was obtained from the Austrian Institute of Technology GmbH (Monika Schmoll; Tulln, Austria), and *T. atrobrunneum* was isolated from a wood sample in 2018 (Bavaria, Germany). Both strains were identified on a molecular level following Cai & Druzhinina [15] and tested in vitro for their biocontrol capacities [117]. As potential preys the ECM basidiomycetes *Laccaria bicolor* S238N (Institute National de la Recherche Agronomique, Nancy, France) and *Hebeloma cylindrosporum* (Technical University Dresden, Germany) and the plant pathogens *Fusarium graminearum* PH-1 (University of Hamburg, Germany) and *Alternaria alternata* 22-2 (Phytopathology, Technical University of Munich) were used. Agar plugs from ECM fungi and *F. graminearum,* and spores from *Trichoderma* spp. and *A. alternata* were routinely sub-cultured on potato dextrose agar and incubated at 21 °C and 75% humidity in constant darkness.

### Bio-fertilizer and biocontrol capacity in *P. x canescens*

*Populus* x *canescens* INRA clone 717 1-B4 was micropropagated routinely in Schenk and Hildebrandt medium (SH medium, [108]) as described by [7] and [82] and cultivated at 21 °C, 75% humidity, and 16 h photoperiod with 105 µmol^−2^ s^−1^ (daylight white color 865). Poplar micro-cuttings were transferred to SH medium to induce rooting and after four weeks plants with similar height and root length were selected and transferred into autoclaved jars (RR80, J. WECK GmbH & Co KG, Wehr-Öflingen, Germany) filled with 200 ml substrate (60% vermiculite (1904, Jungepflanzen, Forchheim, Germany), 20% fine sand (0.71–1.25 mm particle size, MGS Shopping, Hohenthann, Germany), 20% perlite (KPP, Knauf, Iphofen, Germany)). The jars were sealed with a transparent gas and water permeable membrane (Z380059, Breathe-Easy®, Sigma, Deisenhofen, Germany) (Additional file Fig. S1). Test plants were inoculated with five ml spore suspension of *T. harzianum* WM24a1 and *T. atrobrunneum* containing 10^6^ spores ml^−1^ in sterile water and the control plants were mock inoculated with sterile water. Directly after transfer and every two weeks plants were watered with ten ml of ¼ strength Long Ashton nutrient solution (Hewitt & Smith, 1975). The 5-week-old plants were inoculated again with five ml of a spore suspension containing 1 × 10^6^ spores ml^−1^. After four days, one leaf per plant was wounded at four sites with a sterile needle. For infection with *A. alternata* five µl of a spore solution containing 3 × 10^6^ spores ml^−1^ was pipetted directly to the wounding site. For mock inoculation autoclaved water without spores was used and five replicates for each treatment were prepared. Pictures of the leaves were taken after five days and the total infection area per leaf was measured using ImageJ v1.53e software (Wayne Rasby, National Institute of Health, USA, http://imageJ.nih.gov/ij). Plant height, leaf number and shoot and root fresh weight were assessed for a subset of plants (*n* = 7) after 6 weeks. Dry weight was determined after 48 h at 50 °C.

### Antagonistic activity of *Trichoderma*

For in vitro antagonism assays in dual culture, the second fungal partners *H. cylindrosporum*, *L. bicolor*, *F. graminearum,* or *A. alternata* were inoculated on solid Modified Melin-Norkrans synthetic medium (MMN) [81, 82] in non-split and split Petri dishes [36] to also investigate the involvement of fungal VOCs during mycoparasitic interactions. ECM were incubated for two weeks and the pathogens one week, to compensate the slower growth of ECM. *Trichoderma* strains were inoculated on the opposite side of the plate and after three days photos were taken. The colony area (cm^2^) of fungal mycelium in media contact (MC) and air contact (AC) was measured using ImageJ v1.53e software. As a control, each fungus was grown alone. The inhibitory effect was calculated by using the following formula [99]:

$$\text{Change in colony area compared to control }\left(\text{\%}\right)= \frac{\text{D}1-\text{D}2}{\text{D}1}\times 100$$

With D1 = colony area of control condition and D2 = colony area of confrontation.

### Physiological response of *Trichoderma* using race tube system

The olfactometer “race tube”-like system (Additional file Fig. S3, Methods S1) was composed of two 220 ml sample cups (391-0023, VWR, Darmstadt, Germany) and a 50 ml serological pipette (612-3696, VWR, Darmstadt, Germany). Fungal partners were inoculated into the right tube and *Trichoderma* was inoculated with spore solution into the middle of the race tube. For AC condition the second fungus was inoculated into a small petri dish and placed into the test cup. The left control tube remained uninoculated. Hyphal growth of *Trichoderma* was monitored at 48 h, 72 h, 96 h, 120 h, and 144 h, respectively, after inoculation. As a control, *Trichoderma* was challenged with itself. In contrast to plate assays, which lack the capability for directional growth selection, the new system facilitates investigations into interactions spanning longer distances, enabling a choice of growth direction, as it would be the case under most natural conditions. The direction of hyphal growth of *Trichoderma* was determined by the percentage of total growth between the described time points and ∆ % of growth was calculated with the following formula:

$$\Delta \text{ growth direction }\left[\text{\%}\right]=\text{ \% of total growth towards partner}-\text{\% of total growth towards control}$$

### Transcriptomic analysis of confrontations with phytopathogens and ectomycorrhiza

To determine whether the presence of a plant-beneficial and a plant-pathogenic prey affects the expression of mycoparasitism-related genes during different degrees of contact, an RNA-Seq experiment was performed. Confrontation scenarios where the same as for testing antagonistic activity. Fungi were inoculated on modified MMN media overlaid with wet autoclaved cellophane membrane (Natureflex 32 g m^−2^, HERA, Schotten, Germany) to enable biomass harvest without agar residues at three and six days after *Trichoderma* inoculation. For RNA extraction fungal biomass was scraped with a sterile spatula from the interaction zone and immediately ground in liquid nitrogen and stored at −80 °C till further processing. RNA was extracted from four biological replicates using the kit NucleoSpin RNA Plant and Fungi (Machery-Nagel, Düren, Germany) following the manufacturer’s instructions. Integrity and total amount of RNA was detected by bioanalyzer and Qubit (RNA High Sensitivity assay, Aligent Technologies, Santa Clara, USA).

Qualified RNA was subjected to cDNA library preparation using the Illumina stranded mRNA-Kit (Illumina, San Diego, USA) quantified and qualified (DNA High Sensitivity assay, Aligent Technologies, Santa Clara, USA) by the chair of Animal Physiology and Immunology at TUM. Sequencing of barcoded libraries was done at IMGM laboratories (Martinsried, Germany) with NovaSeq 6000 for 100 bp single-end reads to a depth of 18 million reads per sample. *H. cylindrosporum* could not be included in this transcriptomic analysis, since it was not available at that time.

### Bioinformatic analysis

Sequencing data was processed using nf-core/rna-seq v3.12.0 workflow [27] and executed with Nextflow v23.10.0 [23]. The reads were trimmed using Cutadapt (v4.8) [74] to remove poly-A tails, adaptor sequence contaminations and low-quality bases and aligned to the concatenated reference genomes with STAR [24]. Gene-level read counts were determined using Salmon [90] and subjected to downstream analysis. Differential gene expression analysis was conducted using the DESeq2 R package (v1.16.1) to normalize the libraries based on the geometric mean of the read counts and then calculate the log2fold change (LFC) between the experimental test conditions and the control condition [68]. Genes were identified to be differentially expressed (DEGs) with adjusted *p*-value (FDR) < 0.01. Upregulated DEGs (LFC > 0) were submitted to FungiFun 2.2.8 BETA [95] with *T.* *harzianum* CBS 226.95 and *L. bicolor* S238N-H82 / ATCC MYA-4686 as reference species. DEGs were classified based on Functional Catalogue (FunCat) [104], Gene Ontology (GO) [50], and Kyoto Encyclopedia of Genes and Genomes (KEGG) [57] and tested for enrichment (adj. *p*-value < 0.05). Presence of signal peptides in DEGs was predicted with SignalP 5.0 [3]. Self-organizing tree algorithm (SOTA) was used to cluster common DEGs based on expression patterns between the different test conditions using Pearson’s correlation and MeV v4.4.1 [54].

### Statistical analysis and visualization

Data was processed for statistical data analysis with OriginPro (v.2022b, OriginLab Corporation, Northampton, USA) and tested for normal distribution (Shapiro–Wilk test), and for variance homogeneity (Levene’s test). One-way ANOVA combined with Bonferroni test or Student’s *t*-test was applied for pairwise comparison. For plant data, one-way ANOVA was applied combined with Fisher’s least significant difference (LSD) test. Figures were created with BioRender.com (license WN26OFV8MS).

## Results

### *Trichoderma* spp. are displaying bio-fertilizer and biocontrol capacity in grey poplar

**Fig. 1:**
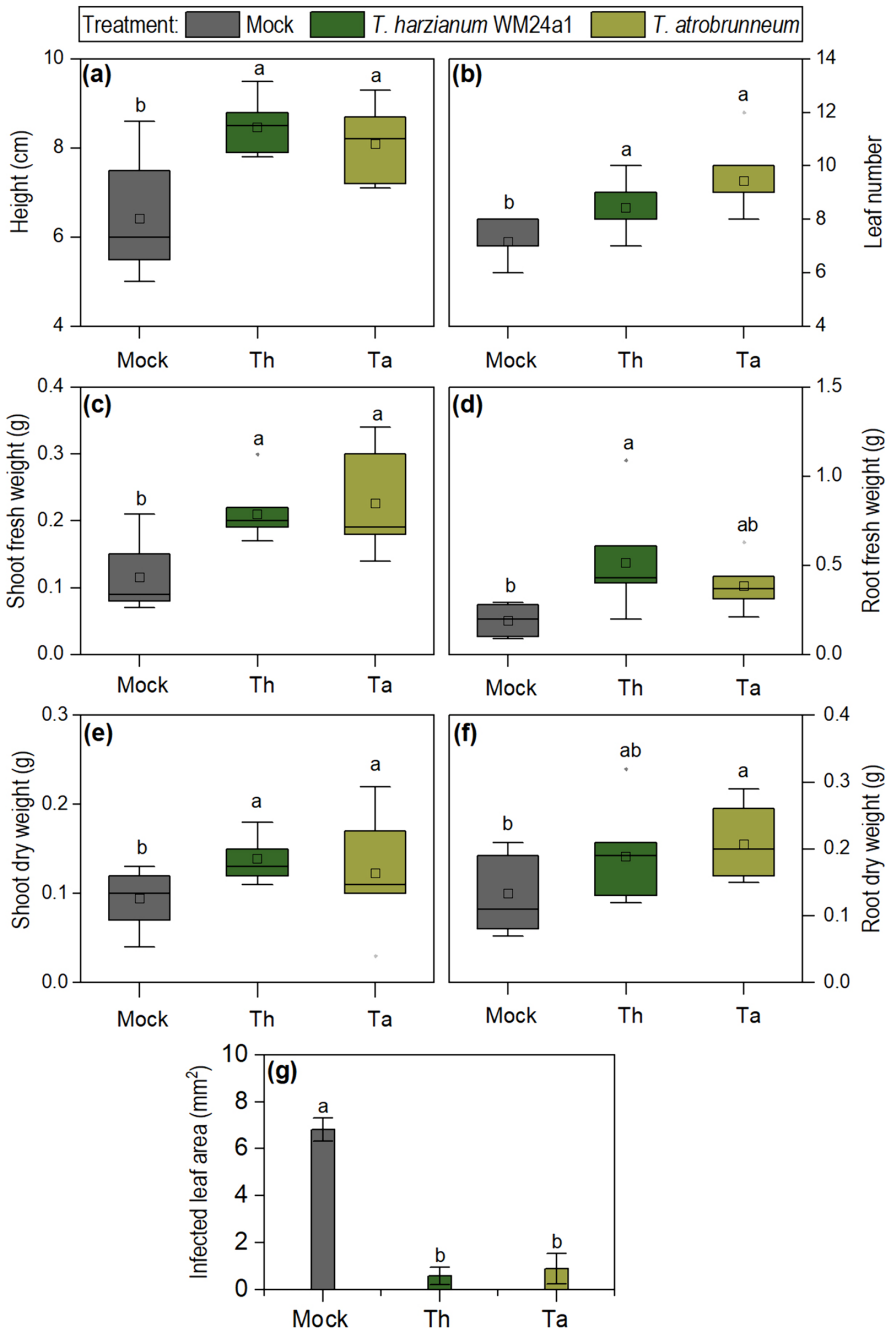
Evaluation of bio-fertilizer and biocontrol capacity of *T. harzianum* WM24a1 and *T. atrobrunneum* in *Populus* x *canescens*. Plant height (**a**), leaf number (**b**), shoot fresh and dry weight **(c**, **e)** and root fresh and dry weight (**d**, **f**) were assessed after six weeks of cultivation (*n* = 7). A subset of plants was inoculated again with 5 ml spore solutions containing 10^6^ spores ml^−1^. After 4 days one leaf per plant was injured with a sterile needle and infected with *A. alternata* spore solution (*n* = 5). Infection area in mm.^2^ (**g**). Significances were determined by one-way ANOVA and LSD, p < 0.05

When considering *Trichoderma* strains as a BCA for poplar plantations, the potential to induce effective plant systemic resistance may vary between different strains used. It is therefore important to exclude potential negative effects by evaluating the interaction of the biocontrol strain and the plant. The biofertilizer capacity was evaluated by comparing plant height, leaf number, shoot and root fresh and dry weight to the untreated control plants (Fig. 1). The chosen strains significantly increased plant height, average leaf number (from 7.2 (± 1.6) to 8.4 (± 0.9) with *T. harzianum* treatment and 9.4 (± 1.2) for *T. atrobrunneum* treatment) (Fig. 1a, b), as well as shoot and root fresh weight (Fig. 1c, d). To evaluate the potential biocontrol capacity of both strains*,* one leaf of each poplar-plant was injured and infected with spores of *A. alternata*. Both *Trichoderma* strains significantly decreased signs of infection in leaves (Fig. 1g) reflecting a positive influence on the induced systemic resistance in *P.* x *canescens.*

### Differences in antagonistic activity of *Trichoderma* towards plant-pathogenic and ECM fungi

To examine the potential inhibitory effect of *Trichoderma* species towards plant-pathogenic and plant-beneficial fungi, we initially set up dual confrontation assays with *F. graminearum*, *A. alternata, L. bicolor* and *H. cylindrosporum* in different degrees of contact in standard Petri dish systems (Additional file Fig. S2).

**Fig. 2:**
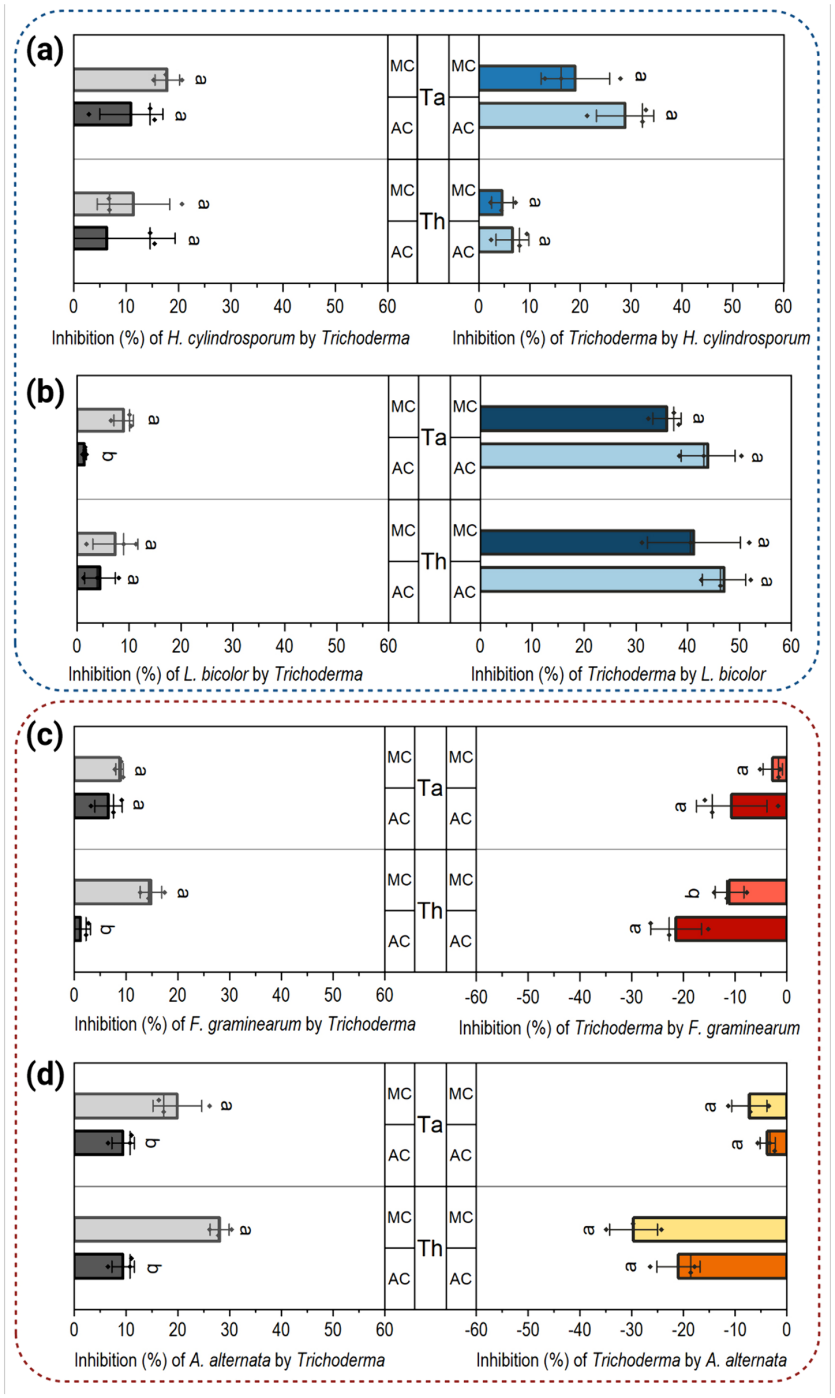
Growth inhibition during co-cultivation of *Trichoderma* spp. with the two ectomycorrhizal fungi *H. cylindrosporum* **(a)** and *L. bicolor* **(b)** and the two plant-pathogenic fungi *F. graminearum* **(c)** and *A. alternata* **(d)**. Growth inhibition of each fungus during co-cultivations was calculated by comparing the colony area with single controls for MC and AC three days after inoculation with *Trichoderma*. Significances were determined for each contact stage separately with Student’s *t*-test, *p* < 0.05, *n* = 3

A clear inhibitory effect of both *Trichoderma* strains towards all confrontation partners was observed three days after inoculation with *Trichoderma*, albeit differing in severity depending on the partner and being generally stronger during conditions that allowed media contact (MC), compared to the situation in a split-plate that allowed only contact through the headspace (air contact; AC). The inhibitory effect of both *Trichoderma* strains was stronger towards *H. cylindrosporum* compared to *L. bicolor* (Fig. 2a, b). The presence of *H. cylindrosporum* and *L.* *bicolor*, on the other hand, was found to inhibit the growth of both *Trichoderma* strains (between 4 and 47%), and this effect was more pronounced, albeit not significantly, in the AC conditions and somewhat more robust for *L. bicolor* than for *H. cylindrosporum* (Fig. 2a, b). Also, *T. harzianum* showed a stronger inhibitory effect on both pathogens during MC compared to AC, whereas *T. atrobrunneum* only showed stronger inhibitory effect during MC towards *A. alternata* (Fig. 2c, d). At the same time, the presence of both pathogens led to an enhanced colony diameter of *Trichoderma*, indicated by negative values (Fig. 2c, d). In *T. atrobrunneum*, this effect ranged between 2 and 10% and in *T. harzianum* between 11 and 29% compared to the *Trichoderma*-only control. Intriguingly, while this increase was stronger during AC confrontation with *F. graminearum* compared to MC confrontation, no significant differences could be detected between AC and MC confrontation with *A. alternata*. Overall, these data show that while the ECM fungi have a robust repelling effect on *Trichoderma* spp., as indicated by decreased colony growth (Fig. 2a, b), *Trichoderma* spp. seem to be attracted to plant-pathogenic fungi, as indicated by increased colony growth (Fig. 2c, d).

### A novel olfactometer “race tube”-like system to quantify the directional growth response to fungal partners

**Fig. 3:**
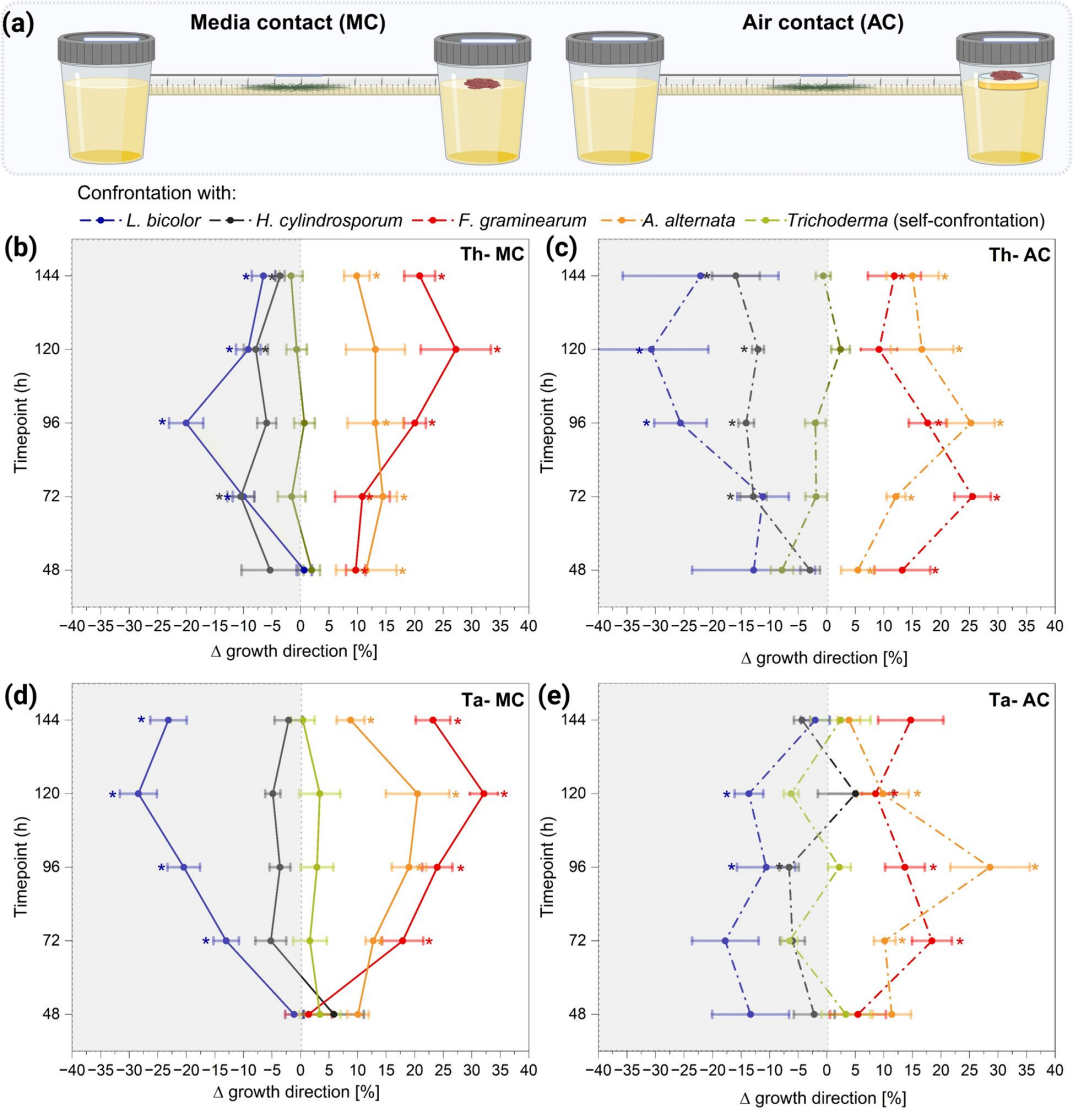
Experimental setup of olfactometer “race tube”-like system and the confrontations with fungal partner during media (MC) and air contact (AC) **(a)**. Physiological response of *T. harzianum* WM24a1 **(b**, **c)** and *T. atrobrunneum* **(d**, **e)** in the presence of *H. cylindrosporum* (grey), *L bicolor* (blue), *F. graminearum* (red) and *A. alternata* (orange). The direction of growth was determined as ∆ growth direction (%) between the different time points. As a control, *Trichoderma* was challenged with itself (green). Significances were determined with Student’s *t*-test compared to the self-confrontation, *p* < 0.05, *n* = 5

To overcome the limitations of more traditional plate confrontation assays, we developed a novel olfactometer “race-tube”-like system. Differing from the petri dish system, this experimental setup allows for a two-way choice of growth direction and therefore an observation of the directed growth of *Trichoderma* in presence of a second fungus (Fig. 3a). Furthermore, the growth direction can be quantified over longer distances and time and therefore in a much more reliable fashion. Self-confrontation of both *Trichoderma* strains led to Δ growth direction values near 0, indicating an equal growth towards both directions (Fig. 3b–e). Using the new system, we could confirm the overall effects observed in the plate-based system. However, it became obvious that both *Trichoderma* strains were not simply inhibited by the ECM fungi, but indeed chose to grow away from them, visible by stronger growth in the opposite direction, leading to negative Δ growth direction values. This effect was stronger for *T. harzianum* during AC compared to MC (Fig. 3b, c), whereas the opposite was observed for *T. atrobrunneum* (Fig. 3d). The situation was found to be completely different in presence of phytopathogens, and both *Trichoderma* strains displayed a clear growth preference towards those fungi, as indicated by positive Δ growth direction values (Fig. 3b–e). In *T.* *harzianum* this effect was stronger during the first 72 h in AC compared to MC, shifting to more pronounced effects in MC compared to AC after 96 h (Fig. 3b, c). Interestingly, in both *Trichoderma* strains the directed growth towards the pathogens in AC confrontation decreased slightly after 96 h, and in MC confrontation after 120 h. Overall, the physiological responses indicated a negative chemotropism in presence of ECM and a positive chemotropism in presence of phytopathogens taking effect already at comparably long distances.

### Distinct patterns in *Trichoderma* global gene expression

**Fig. 4:**
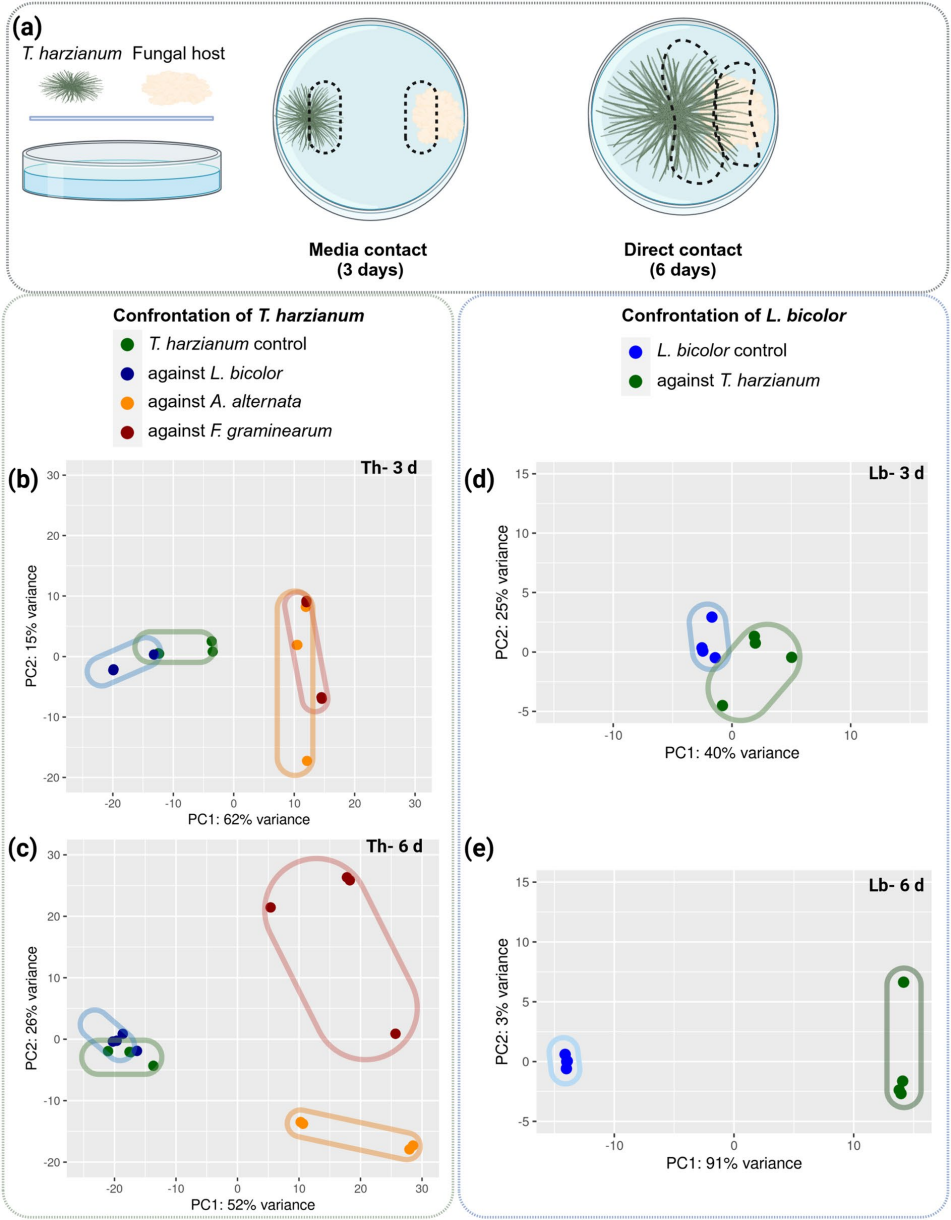
Transcriptomic analysis of fungal confrontations. Experimental setup alongside with depicting areas of biomass harvest from the zone of interaction **(a)**. PCA plots illustrate differential gene expression in *T. harzianum* after three (MC) and six days (DC) **(b**, **c)** and in *L. bicolor* **(d**, **e)** based on the top 500 differentially expressed genes among all conditions

To identify DEGs related to the strongly differing reaction of *Trichoderma* to ECM and phytopathogenic fungi, another series of plate-based confrontations was conducted with *T.* *harzianum* on one side and *L. bicolor*, *F. graminearum*, or *A. alternata* on the other. The plate system was used in this experiment, since it allowed harvesting biomass directly from the zone of interaction (Fig. 4a). Samples were taken three days after inoculation before any kind of physical contact (MC) and six days after inoculation when a direct hyphal interaction was established (DC).

Principal component analysis (PCA) revealed clear cluster formation for *Trichoderma*-only control, *Trichoderma* confronting pathogens and *Trichoderma* confronting *L. bicolor*. Thereby the pathogen-confrontations clustered away, while confrontation with *L. bicolor* clustered close to the control, (Fig. 4b). At DC-phase, the pathogen interactions separated into individual clusters, while the confrontation with *L. bicolor* still clustered with the control (Fig. 4c). This differential clustering highlights the distinct expression patterns during the confrontation with the pathogens while interaction with *L. bicolor* resulted in only modest changes.

Distinct patterns across the confrontations with the ECM and the pathogens during MC emerged also when looking at the gene expression more closely (Additional file Fig. **S4**). The confrontation with *L. bicolor* was characterized by small LFC values compared to the control, representing minimal changes in gene expression. To nevertheless ensure not to lose potentially relevant genes in the downstream analysis, we employed a threshold of adjusted FDR < 0.01 without an additional LFC threshold. Especially during the MC stage without physical contact, we expected the potential signaling mechanisms not to display extreme fold-changes, which should nevertheless be significant. This approach allowed to capture the nuanced variations in gene expression during confrontation with the ECM. Conversely, in confrontation with the pathogens during the MC, the volcano plots illustrate a broader dispersion of points with higher LFC values, indicating a notable and pronounced alteration in gene expression.

### Unique genetic response of *T. harzianum* towards *L. bicolor*

**Fig. 5:**
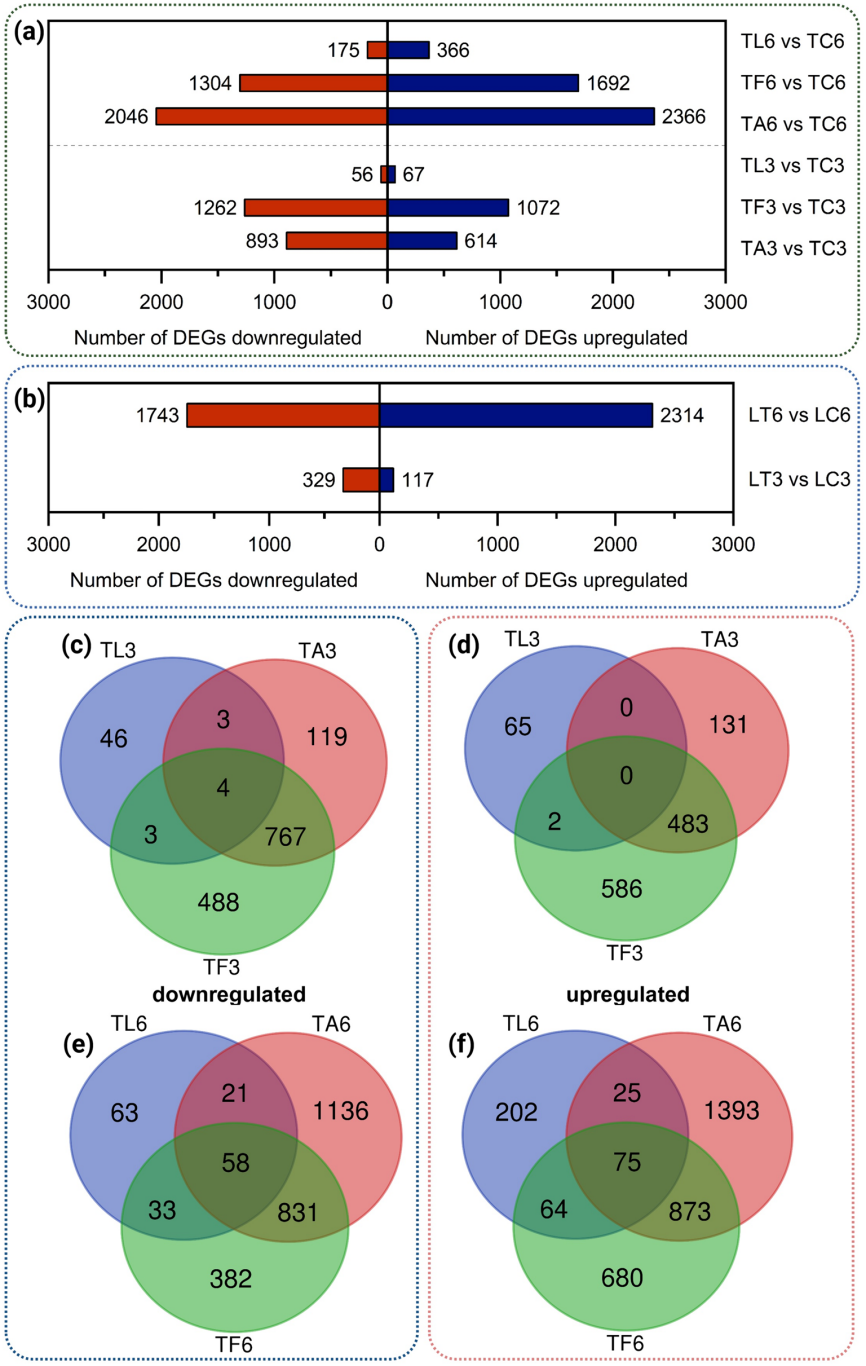
Number of DEGs (FDR < 0.01) that were up- or downregulated in the confrontation of *T. harzianum* with *L. bicolor* (TL), *A. alternata* (TA), and *F. graminearum* (TF) compared to the control condition after three days (TL3, TA3, TF3) and six days (TL6, TA6, TF6) **(a)**. Number of DEGs (FDR < 0.01) that were up- or downregulated in the confrontation of *L.* *bicolor* with *T.* *harzianum* (LT) after three and six days (LT3 and LT6, respectively) **(b)**. Venn diagrams showing common and unique DEGs among the three different confrontations of *T.* *harzianum* with *L.* *bicolor* (TL), *A. alternata* (TA), and *F. graminearum* (TF) after three days **(c**, **d)** and after six days **(e**, **f)**

Overall, the interaction of *T. harzianum* and *L. bicolor* during MC (three days) led to the up- and downregulation of only 67 and 56 DEGs in *T. harzianum*, respectively (Fig. 5a**,** Additional file Table S1). This observation aligns with other present observations. The changes in the transcriptome of *T.* *harzianum* were small compared to the distinct and pronounced changes during confrontation with *F. graminearum* and *A. alternata*, which led to 614 and 1,072 upregulated DEGs and 1,262 and 893 downregulated DEGs, respectively. During DC (day six) 366 and 175 genes were significantly up- and downregulated in *T.* *harzianum* during interaction with *L. bicolor*, whereas the presence of both pathogens led to much more DEGs (2,366 and 1,692 upregulated DEGs during confrontation with *A.* *alternata* and *F.* *graminearum*, respectively) (Fig. 5a). After three days of confrontation with the two pathogens, 767 and 483 DEGs were commonly up- and downregulated in *T.* *harzianum* (Fig. 5c, d), while during confrontation with *L. bicolor* (TL3), 65 and 46 were uniquely up- and downregulated, respectively (Supplementary Table S2). During DC (six days), confrontation with pathogens up- and downregulated 873 and 831 shared DEGs in *T.* *harzianum*, while only 75 and 58 DEGs were common also in the confrontation with *L.* *bicolor* (Fig. 5e, f).

During MC (three days), at least 13 genes from among the common and most upregulated 30 DEGs in confrontation with *A. alternata* and *F. graminearum* are known to play a crucial role in the process of mycoparasitism, clearly showing induction of related gene cascades already long before direct hyphal contact. On the contrary, during confrontation with *L. bicolor* those mycoparasitism-related genes rather showed a downregulation (Table 1). Interestingly, a terpene synthase (M431DRAFT_113113; LFC −0.9) was significantly downregulated as well. Furthermore, two short and uncharacterized signal peptide-containing proteins (M431DRAFT_69921, M431DRAFT_129453) were significantly induced.

**Table 1:** Genes in *T. harzianum* with known or predicted functions in the process of mycoparasitism that were found to be upregulated during MC in presence of *A. alternata* (TA3) and *F. graminearum* (TF3), but not *L. bicolor* (TL3), at three days post inoculation

Predicted protein name	Gene ID	log2FC TA3	log2FC TF3	log2FC TL3	Assigned GO terms
glucan endo-1,3-beta-D-glucosidase^†^	M431DRAFT_479664	***2.84***	***4.50***	*0.13*	Beta-glucosidase activity [GO:0008422]; Polysaccharide catabolic process [GO:0000272]
Carbohydrate-binding module family 24 protein^†^	M431DRAFT_525334	***1.87***	***3.56***	**−** **0.53**	Glucan endo-1,3-alpha-glucosidase activity [GO:0051118]
Oligopeptide transporter	M431DRAFT_529850	***2.45***	***3.51***	**−** **0.67**	Oligopeptide transmembrane transporter activity [GO:0035673]; membrane [GO:0016020]
Peptidase S1 domain-containing protein^†^	M431DRAFT_526221	***2.13***	***3.37***	**−** **0.62**	Serine-type endopeptidase activity [GO:0004252]; Proteolysis [GO:0006508]
Glycoside hydrolase family 18 protein^†^	M431DRAFT_509593	***2.48***	***3.30***	**−** **0.23**	Hydrolase activity [GO:0016787]; Carbohydrate metabolic process [GO:0005975]
Carbohydrate-binding module family 13 protein	M431DRAFT_9084	***1.73***	***2.17***	**−** **0.35**	Carbohydrate metabolic process [GO:0005975]
Glycoside hydrolase family 3 protein, β-glucosidase^†^	M431DRAFT_504078	***1.65***	***1.97***	**−** **0.51**	Hydrolase activity, hydrolyzing O-glycosyl compounds [GO:0004553]; Polysaccharide catabolic process [GO:0000272]
Chitinase^†^	M431DRAFT_505895	***1.46***	***1.95***	**−** **0.32**	Chitin binding [GO:0008061]; Chitinase activity [GO:0004568]; Chitin catabolic process [GO:0006032]; Polysaccharide catabolic process [GO:0000272]
Chitinase^†^	M431DRAFT_500888	***2.30***	***1.89***	**NA**	Cellulose binding [GO:0030248]; Chitinase activity [GO:0004568]; Chitin catabolic process [GO:0006032]; Polysaccharide catabolic process [GO:0000272]
Amino acid permease/ SLC12A domain-containing protein	M431DRAFT_92558	***1.07***	***1.55***	**−** **0.44**	Membrane [GO:0016020]; Amino acid transport [GO:0006865]; Transmembrane transport [GO:0055085]
Major facilitator superfamily (MFS) profile domain-containing protein	M431DRAFT_101977	***1.65***	***1.52***	*0.84*	Transmembrane transporter activity [GO:0022857]; Membrane [GO:0016020]
Glycosyl-transferase family 32 protein	M431DRAFT_238777	*0.94*	***1.49***	**−** **0.17**	Alpha-1,6-mannosyltransferase activity [GO:0000009]; Carbohydrate derivative metabolic process [GO:1901135]
Chitinase^†^	M431DRAFT_517960	*0.74*	***1.37***	*0.05*	Chitin binding [GO:0008061]; Chitinase activity [GO:0004568]; Chitin catabolic process [GO:0006032]; Polysaccharide catabolic process [GO:0000272]

Predicted protein names were retrieved from UniProt and log2 fold-changes (log2FC) of all three confrontations are displayed and highlighted Bold for log2FC < 0, Italic 0 > log2FC > 1 and Bold, Italic for log2FC > 1. Genes which were removed due to low counts show log2FC of NA. DEGs containing a signal-peptide predicted by SignalP are marked with a^†^

**Fig. 6:**
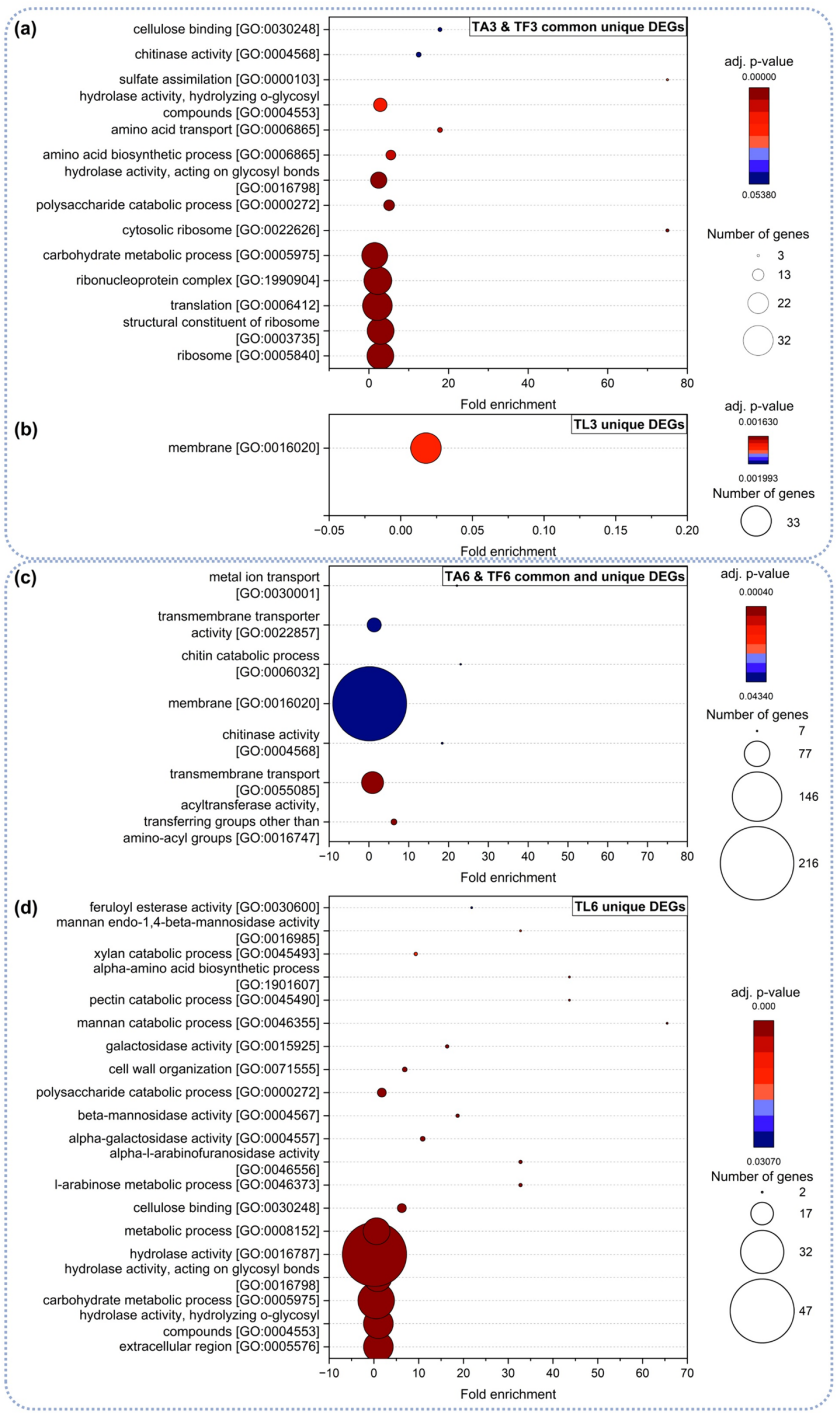
Gene ontology enrichment analysis of common and unique upregulated DEGs in *T. harzianum* during the interaction with *A. alternata* (TA3, TA6) and *F. graminearum* (TF3, TF6) after three days (MC) **(a)** and six days (DC) **(c)**. GO enrichment analysis of DEGs uniquely upregulated in *T. harzianum* in presence of *L. bicolor* after three days (TL3) **(b)** and six days (TL6) **(d)**. GO terms were assumed to be significantly enriched with adjusted *p*-value < 0.05

Gene ontology analysis of upregulated genes showed clear enrichment (adj. *p*-value < 0.05) of terms involved in primary metabolic activity, such as “Ribosomes”, “Translation” and “Carbohydrate metabolic process” (Fig. 6a). This aligns with the phenotypic observations of increased colony area when *T. harzianum* was confronted with plant pathogens. Furthermore, GO terms of “Cellulose binding”, “Chitinase activity” and “Hydrolase activity” were significantly enriched, corroborating that *Trichoderma* is able to sense potential prey already before direct hyphal contact. Conversely, GO enrichment analysis of upregulated DEGs in interaction with *L. bicolor* revealed a much more limited outcome with membrane-related annotation being the only enriched category (Fig. 6b).

**Fig. 7:**
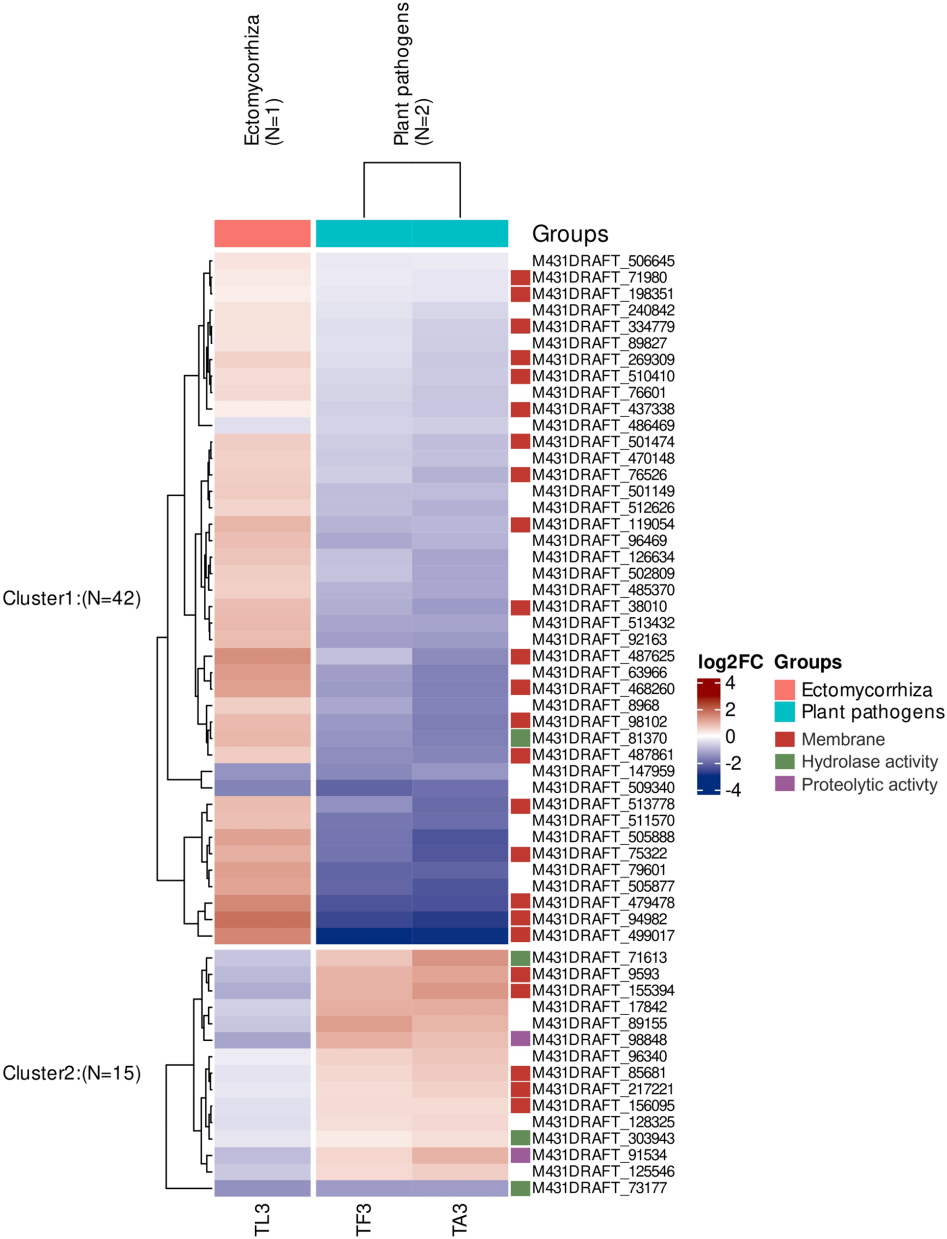
Heatmap of the 57 common DEGs in *T. harzianum* after three days during confrontation with *L. bicolor* (TL3), *A. alternata* (TA3) and *F. graminearum* (TF3) shown as log2 fold change (normalized to single control of *T. harzianum* TC3) and clustered based on expression using SOTA

Intriguingly, we identified 57 genes that were differentially expressed in all three test conditions during MC (three days), but which nevertheless show distinct expression patterns, according to the lifestyles. Expression profiles of those common DEGs by SOTA method using Pearson’s correlation revealed two distinct clusters (Fig. 7, Additional files Fig. S5, Table S3): 42 genes (cluster 1) showed a significant induction in *T. harzianum* during confrontation with *L. bicolor* and significant repression during confrontation with the pathogens. The remaining 15 DEGs (cluster 2), showed an opposite regulation, being significantly upregulated during the confrontation with the pathogens and downregulated during confrontation with *L.* *bicolor*. Several DEGs in cluster 1 are assigned to GO terms of “Membrane” and associated with transport activities, as seen above. Interestingly, also a small secreted protein (M431DRAFT_96469; signal peptide likelihood: 0.99) was significantly induced in confrontation with *L. bicolor* with an LFC of 0.83 (FDR 0.001) and downregulated in presence of *A. alternata* and *F. graminearum* with LFC of −0.99 and −1.15 (FDR 2.75E^−8^ and 1.45E^−6^), respectively. Cluster 2 contains several DEGs annotated with GO terms of “Hydrolase activity” and a peptidase A4 family protein upregulated with LFCs of 0.80 and 1.01 (FDR 2.96E^−11^ and 1.23E^−7^) in confrontation with *A.* *alternata* and *F.* *graminearum*, respectively, and downregulated in confrontation with *L.* *bicolor* (LFC of -1.19; FDR 3.7E^−14^).

GO-term enrichment analysis of *T. harzianum* genes upregulated at day six of contact with both pathogens identified “Membrane”-related categories, “Transport”, as well as “Chitin catabolic processes”, as to be expected during mycoparasitism (Fig. 6c). During interaction with the ECM, significantly enriched GO terms were predominantly associated with (extracellular) carbohydrate metabolism and cell wall organization (Fig. 6d).

Overall, many processes related to mycoparasitism were upregulated already in the early stage of interaction with the two pathogens, while the presence of *L. bicolor* did not lead to any strong induction or repression of specific genes. Also during DC, only contact with the plant pathogens led to clearly recognizable antagonistic gene expression patterns.

### Transcriptomic alterations in *L. bicolor *in confrontation with *T. harzianum*

To identify ECM genes involved in the interaction with *Trichoderma,* we next investigated transcriptomic changes in *L. bicolor* during confrontation with *T. harzianum*. The LFC values were similarly small as in *T. harzianum* during interaction with *L. bicolor* (Additional file Fig. S3). PCA analysis of the *L. bicolor* transcriptome revealed very similar expression patterns like in the single fungus controls at three days (MC) and only clear distinct clustering after six days (DC; Fig. 4d, e).

During MC, 117 and 329 DEGs were up- and downregulated, respectively, in *L. bicolor* in confrontation with *T. harzianum* (Fig. 5b). Enrichment analysis of upregulated DEGs after three days revealed enrichment of only one GO term, “DNA binding transcription factor activity”, while no pathways in FunCat or KEGG were significantly enriched (Supplementary Table S4). However, several of the upregulated DEGs were annotated with FunCat main categories “Transcription”, “Metabolism”, “Protein with binding function or cofactor requirement”, and “Cellular communication / signal transduction”. This includes one Nrg1-like Zn-finger transcription factor (LACBIDRAFT_296037) being significantly induced in presence of *T. harzianum*, as well as two SNF2 family DNA-dependent ATPases (LACBIDRAFT_301027, LACBIDRAFT_396054). Notably, three upregulated DEGs (LACBIDRAFT_240638, LACBIDRAFT_246709, LACBIDRAFT_248257) were linked to “G-protein coupled receptor signalling, Cellular communication / signal transduction mechanism” pathways, indicating the initiation of signaling cascades in *L. bicolor* as potential response to signaling molecules derived by *T.* *harzianum*. Moreover, several of the 117 upregulated DEGs were assigned to subcategories of the KEGG main pathways “Metabolism”, such as “Biosynthesis of secondary metabolites”, “Steroid biosynthesis”, “alpha-Linolenic acid metabolism”, “Terpenoid backbone biosynthesis”, and “Fatty acid metabolism”.

Interestingly, the most upregulated gene, LACBIDRAFT_304386 (LFC 0.86), was predicted to be a signal peptide-containing protein (likelihood of 0.99). The third most upregulated DEG was an oligopeptide transporter (LACBIDRAFT_302225; LFC 0.71), suggesting increased fluxes of metabolites. A slightly upregulated signal peptide-containing (likelihood 0.94) tripeptidyl-peptidase II (LACBIDRAFT_191088) might indicate a very moderate triggering of defense mechanisms. Moreover, a plasma membrane fusion protein (LACBIDRAFT_231982), part of the fusion machinery and involved in stabilizing the plasma membrane (“Mating projection tip”; “Plasma membrane”) was increased by 39%, which could be indicating a reaction of cell wall and membrane remodeling in response to secreted enzymes or effector proteins by *T. harzianum*.

**Fig. 8:**
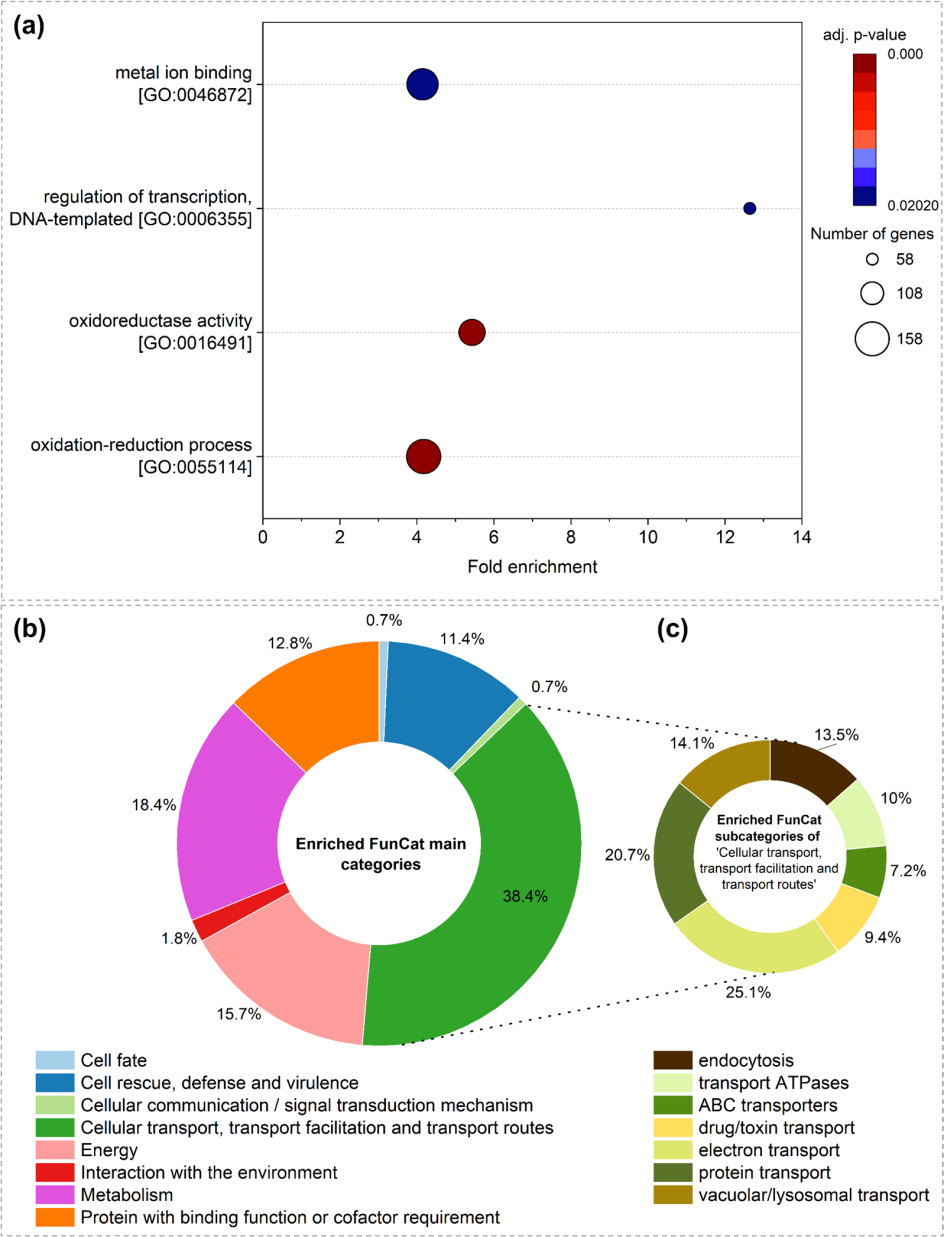
Gene ontology enrichment analysis of upregulated DEGs in *L. bicolor* in presence of *T. harzianum* after six days of co-cultivation (DC) **(a)**. FunCat enrichment analysis of upregulated DEGs in *L. bicolor* during DC (six days) with *T. harzianum*. Significant enriched FunCat main categories **(b)** and enriched subcategories of “Cellular transport, transport facilitation and transport routes” **(c)**. GO terms and FunCat categories were assumed to be significantly enriched with adjusted *p*-value < 0.05

The DC condition led to a stronger response, with 2314 and 1743 up- and downregulated DEGs (Fig. 5b), respectively. Upregulated DEGs revealed enriched GO terms of “Oxidation–reduction process”, “Oxireductase activity”, “Regulation of transcription”, and “Metal ion binding” (Fig. 8a). From KEGG main category “Metabolism”, the pathways of “Oxidative phosphorylation”, “Citrate cycle (TCA cycle)”, “Valine, leucine and isoleucine degradation”, “Pyruvate metabolism” and “Carbon metabolism” were significantly enriched, as well as FunCat categories such as “Cellular transport, transport facilitation and transport routes” (38.4%) “Metabolism” (18.4%), “Energy” (15.7%), “Protein with binding function of cofactor requirement” (12.8%), “Cell rescue, defense and virulence” (11.4%), and “Interaction with the environment” (1.8%) (Fig. 8b). Among the transport-related categories, FunCat descriptions of “Endoycytosis”, “Drug/toxin transport”, and “ABC transporters” (Fig. 8c) also indicate defense mechanisms. One of those assigned DEGs was a glutathione transferase (LACBIDRAFT_188517; LFC 5), with known function in detoxification, as well as a multidrug resistance-associated ABC transporter (LACBIDRAFT_318236, LFC 2), and a pleiotrophic drug resistance ABC transporter (LACBIDRAFT_314719, LFC 2.5). Furthermore, several Mycorrhiza-induced Small Secreted Proteins (MiSSPs) such as MISSP6.4 (LACBIDRAFT_316998; LFC 5.57), MISSP16.2 (LACBIDRAFT_333197; LFC 1.9), and MISSP22.4 (LACBIDRAFT_303456; LFC 0.48) were significantly upregulated during DC with *T. harzianum*.

## Discussion

Fungi engage in diverse interactions with their environment including nearby organisms. The fungi used in this study (*Trichoderma* spp., *Laccaria* spp., *Hebeloma* spp., *Fusarium* spp., and *Alternaria* spp.) can all be found in the rhizosphere, where they occupy similar environmental niches, while nevertheless having different lifestyles [44].

The two tested *Trichoderma* strains, *T. harzianum* and *T. atrobrunneum*, both displayed bio-fertilizer activities in *P. x canescens* and significantly reduced susceptibility to *A. alternata* infection. This observation is in line with previous studies, which have observed elevated systemic induced resistance and reduced susceptibility against *A. alternata* in poplar trees by e.g. xylanases produced by *Trichoderma* [35, 137]. The favorable findings regarding the two tested *Trichoderma* strains indicate their potential as BCA for poplar trees. However, in natural conditions, the soil would host other fungi that could confer benefits to the plants [45, 122]. The interactions between two fungal species can manifest as either antagonistic, parasitic, neutral, or synergistic, each yielding distinct outcomes, both for the fungi as well as for potentially involved further interaction partners, such as plants [127, 133]. One can assume that it would be in the interest of the plants to maximize the number of plant-beneficial microorganisms in their rhizosphere. *Trichoderma* spp. are particularly interesting in this regard, since mycotrophy is thought to be an ancient trait of the entire genus [25]. However, whether the application of *Trichoderma* comes at the expense of overall fungal biodiversity, including plant-beneficial fungi, or is somewhat selective, is controversial so far. A risk assessment when considering *Trichoderma* spp. as BCA was therefore suggested already early on, requiring to identify the effect on the target (pathogen) population while evaluating negative effects on non-target (native and plant-beneficial) fungal species, which could have detrimental consequences for the host plant [11]. For example, *Trichoderma* species were found to strongly inhibit mycorrhization of black spruce seedlings by *L. bicolor*, suggesting that they can have a significant impact on ECM [120]. Conversely, the application of the *Trichoderma* bio-inoculant ArborGuard^™^ on *Pinus radiata* seedlings did not adversely affect the ECM colonization in a nursery system [78]. Another study investigated the impact of *T. virens* on pre-mycorrhized *Pinus sylvestris* roots. Intriguingly, while a decreased spore germination of *Trichoderma* was monitored in the rhizosphere, indicating the presence of dampening plant- or ECM-derived processes, the overall plant viability was influenced positively [131]. The described inhibitory effect of ECM towards *Trichoderma* spp. was observed across various in vitro and *in planta* experimental setups (e.g. [36, 78, 120, 131]). A similar inhibitory effect of the related ECM *Laccaria laccata* towards *Trichoderma virens* in co-culture was already described by Werner et al. [131]. Moreover, Summerbell [120] reported the absence of typical hyphal structures, such as intensive branching and coiling structures, of a *Trichoderma* sp. towards *L.* *bicolor* during their interaction, and Zadworny et al. [138] also described a decreased colony area of *T. virens* and *T. harzianum* during co-culture with ECM, as well as altered microtubular cytoskeleton structures in the interaction zone of both fungi, with more pronounced effects in the saprotrophic strains, indicating a stress response.

Likely, the variable outcomes of interaction studies are strongly influenced by the experimental setup, such as space and nutrients, which can unintentionally force the interactions into a specific direction. Our results now demonstrate that in the presence of enough space, *Trichoderma* spp. prefer to grow away from ECMs and do not initiate mycoparasitic programs (either at the physiological or molecular level), that occur in the presence of pathogens long before direct physical contact. Based on these results one could hypothesize that regulatory processes have evolved that maximize the number of plant-beneficial interactions in the rhizosphere [9, 10, 77].

### *Trichoderma* are attracted by plant-pathogens and avoid ECM

The dual confrontation assays performed in this study revealed intriguing insights into the response of *Trichoderma* towards fungi of different lifestyles. Co-culture systems are representing a forced competition of two organisms to decide on the fate of the limited resources in confined spaces. Notably, *Trichoderma* strains displayed varying inhibitory effects against *F. graminearum*, *A. alternata*, *H. cylindrosporum,* and *L. bicolor* during different contact conditions. *T. harzianum* exhibited stronger inhibitory effects towards *F. graminearum* and *A. alternata* especially during MC, indicating its robust potential to produce diffusible, non-volatile secondary metabolites having a significant effect on the growth of plant-pathogenic fungi [36],Küçük & Kivanç; [97, 116].

Fungal interactions are facilitated through the exchange of signaling molecules, which are released by one fungal species and subsequently perceived by receptors present in the other participating species [16, 99, 139]. Fungal VOCs are organic chemicals with low molecular weight that originate from metabolic processes within the fungus, evaporate easily at moderate temperature [72] and participate in the communication between fungal species [36, 37, 130, 134]. Several studies suggest that fungal VOC profiles are similar for fungi with comparable lifestyles [26, 28, 37, 38, 81, 100]. Also *Trichoderma* species have been demonstrated to produce several volatiles, as well as receive, and respond to VOCs [36, 37, 55, 71, 86, 98, 102]. It is already well-known that VOCs are pivotal in orchestrating inter-species and inter-kingdom signaling within the rhizosphere, a dynamic environment, where various organisms interact [29, 132]. They exert influence on growth, defense responses, and behavior of other organisms, with some VOCs exhibiting toxic properties [43, 115]. However, the underlying mechanisms behind these effects and perception mechanisms are poorly understood [41, 132].

In line with our results, earlier co-cultivation scenarios of different *Trichoderma* strains, including *T. harzianum* WM24a1, with *L. bicolor* already revealed a stronger inhibitory effect of the ECM on the biocontrol strain than the other way around [36]. The highest VOC emission rate was thereby detected when the two fungi were separated by several cm from each other, indicating VOCs to be an important tool for long-range inter-species communication and recognition. The upregulation of several metabolic KEGG pathways associated to “Biosynthesis of secondary metabolites”, “Steroid biosynthesis”, “alpha-Linolenic acid metabolism”, “Terpenoid backbone biosynthesis”, and “Fatty acid metabolism”, which we now identified in *L. bicolor* in our setup, might reflect the ECM’s response to increased *Trichoderma*-VOC emission rates already at a distance, potentially representing an activation of secondary metabolite-based communication.

The introduced olfactometer “race-tube”-like system now allowed us to observe a strongly accentuated directional growth behavior of *Trichoderma* spp. compared to the conventional plate confrontation assays, demonstrating the positive or negative chemotropism in response to different fungal partners already at a distance and over time. The growth direction is influenced by soluble and volatile molecules that are involved in fungal chemotropism by acting either as a chemoattractant (positive chemotropism) or as a chemorepellent (negative chemotropism) [59, 80]. Our experiments revealed consistent directional growth behavior of both *Trichoderma* strains towards the plant pathogens and away from ECM, even in the absence of soluble biochemicals or metabolites, suggesting a significant role for VOCs in shaping the overall perception and observed physiological response. This underscores the importance of VOCs as potent mediators within the system, exerting a substantial impact on the inter-species interactions. Therefore, the developed olfactometer “race tube”-like system now enables further investigation of VOC-based fungal interactions and perceptions to screen for chemotropic growth.

### Transcriptomic differences in *T. harzianum* and* L. bicolor*

The process of mycoparasitism is initiated by prey recognition, directed chemotrophic growth towards the prey, followed by direct attack through (bio-)chemical and physical mechanisms, ultimately leading to death and nutrient release [18, 47, 79, 118]. Importantly, the process is signal-dependent, relying on specific inter-species recognition mechanisms [79, 107, 140]. As the fungi are growing towards each other, they are constantly sensing their environment, and specific downstream signalling cascades are initiated based on the given abiotic and biotic conditions [6, 53, 124, 139].

Transcriptomic analysis revealed plenty of common DEGs in *T. harzianum* during confrontation with the pathogens, while the confrontation with *L. bicolor* led to distinct—and much more moderate—expression patterns. These observations confirm the lifestyle-specific recognition between the fungi on the molecular level, leading to staging of a rapid and conserved mycoparasitic attack in case of the pathogens and literally a much more “relaxed” response in case of the ECM. *Trichoderma* spp. produce constitutively extracellular chitinases and proteases, leading to enzymatically released chito-oligosaccharides and oligo-peptides in the presence of a potential prey, which are sensed and initiate the expression of mycoparasitism-related genes [8, 13, 19, 25, 62, 111]. During confrontation with *A. alternata* and *F.* *graminearum* three chitinases, belonging to the hydrolase family 18, were upregulated in *T. harzianum* already at distance (three days, MC), while the presence of *L.* *bicolor* did not activate these genes. Additionally, several genes annotated with hydrolase activity, such as an endo-1,3-β-D-glucanase (M431DRAFT_479664) and a peptidase S1 domain-containing protein (M431DRAFT_526221), presumably involved in fungal cell wall degradation and proteolysis, were induced during confrontations with *A. alternata* and *F.* *graminearum*, but downregulated during confrontation with *L. bicolor*. These results are in line with another comparative transcriptomic analysis that revealed a clear upregulation of mycoparasitism-related genes in *T. atroviride*, *T. reesei*, *T. virens,* as well as *T. harzianum* before direct physical contact with a fungal prey [5, 129]. The absence of induction of those genes during confrontation with *L. bicolor* is speaking in favour of a non-aggressive interaction. Nonetheless, the question whether this is the result of a self-governed decision by *Trichoderma* or is enforced by repressive or inhibitory metabolites produced by *L. bicolor* remains open so far. However, *L. bicolor* displayed only minimal transcriptomic changes in this situation, suggesting a lack of aggressive adaptations.

Next to VOCs, signalling molecules and prey-derived oligo-peptides are assumed to act as ligands for G-protein-coupled receptors (GPCRs) that are involved in transduction of extracellular signals to intracellular-signaling networks in fungi [136] and part of the prey-sensing cascade in *Trichoderma* [12, 53, 64, 87, 111, 139]. Signal reception triggers downstream events via signal transduction mechanisms [6, 16], which play a pivotal role in orchestrating the expression of specific sets of genes that govern the ultimate outcome of the interaction between two fungal species [107]. During MC, we identified three upregulated genes (LACBIDRAFT_240638,LACBIDRAFT_246709; LACBIDRAFT_248257 in *L. bicolor* in presence of *T. harzianum*, which are annotated with FunCat categories “GPCR signalling”, “cellular communication”, and “signal transduction mechanism”. Furthermore, the upregulated transcription factor Nrg1 in *L. bicolor*, has a putative function in carbon catabolite repression [21] and is involved in fungal gene regulation of stress-response to salt and oxidative stress [106].

In *T. harzianum*, furthermore, a rhodopsin domain-containing protein (M431DRAFT_155394) belonging to the GPCR rhodopsin family A [49], was significantly downregulated in presence of *L. bicolor* and induced during confrontation with the pathogens. Additionally, a 3’,5’-cyclic-nucleotide phosphodiesterase (M431DRAFT_74093) was found to be significantly upregulated in *T. harzianum* during MC with *L. bicolor*, indicating modulation of intracellular levels of cyclic nucleotides, such as cyclic adenosine 3’,5’ monophosphate (cAMP). Those messenger molecules are synthesized from ATP by adenylate cyclase activity and activate the cAMP-dependent protein kinase, leading to gene expression regulation by phosphorylation of e.g. transcription factors [121]. Biogenic VOCs emitted by post-harvested tomatoes, specifically ethylene and benzaldehyde, were identified as active compounds found to be tightly bound to GPCRs in *B. cinerea,* leading to a lack of signal transduction to the cAMP pathway, resulting in reduced pathogenicity [64]. The detected differential regulation of signal transduction-related genes in *L. bicolor* and *T. harzianum*, as well as the altered VOC emission profiles observed by Guo et al. [36] during co-cultivation emphasize a VOC-mediated inter-species interaction and GPCRs signal transduction, with a distinct outcome of negative chemotropism and no induction of mycoparasitism-related cascades in *T. harzianum*.

The absence of mycoparasitic activity in *T. harzianum* when encountering ECM may be due to the intricate and specific nature of its host identification mechanisms [5, 80]. The distinction between ECM, which are basidiomycetes, and the ascomycete plant pathogens tested, might be simplified by the large phylogenetic distance between these groups. Nonetheless, *Trichoderma* spp. have been shown to display clear antagonistic behaviour towards wood-decaying basidiomycetes as well [67, 101]. Future studies, including additional strains with more lifestyle and phylum combinations, will help to identify the key compounds (info-chemicals) that allow *Trichoderma* to distinguish between friend and foe.

Several signal-peptide containing proteins have been described as effectors in beneficial plant-fungus interactions, [92], as well as in fungus-fungus interspecies interactions [30], although not all secreted proteins function in this capacity. Interestingly, we identified the unique upregulation of a small-secreted protein (SSP) (M431DRAFT_96469) in *T. harzianum* in the presence of *L. bicolor*, while it was downregulated during confrontation with *A. alternata* and *F. graminearum* during MC. This SSP is a homologue (> 90% sequence identity) of the cysteine-rich effector Tsp1 in *T. virens,* which was found to be induced in the presence of maize [61] and banana roots [83], indicating an involvement in *Trichoderma*-plant interaction and plant defence modulation. Gupta et al. [39] analysed the function of Tsp1 in *T. virens* and identified structural similarity with the two fungal effector proteins PevD1 [14] and Alt a1 [20], which are both interacting with plant defence proteins. The upregulation of this effector in *T. harzianum* confrontation with *L. bicolor* is a new finding and suggests an additional involvement in fungal inter-species interactions.

Also, in *L. bicolor* several short signal peptide-containing proteins and MiSSPs, which might modulate the interaction with *T. harzianum* by acting as secreted effector proteins, were significantly upregulated during confrontation. While ECM fungi are characterized by a restricted number of carbohydrate-active enzymes, their secretomes are enriched in SSPs [91]. Further studies are needed to investigate the role of those detected and potentially secreted proteins in *L. bicolor*. Moreover, considering the plant roots as holobiont, further investigation into the influence of the host plant on the interaction of *T. harzianum* and *L. bicolor* on the mycorrhized roots is required to gain insights into the fungus-fungus signaling mechanisms and the influence of the plant on the overall outcome of the tripartite interaction.

## Conclusions

Concluding, we explored the complex interactions between *Trichoderma* spp. and fungal partners of different lifestyles, including two plant-beneficial ectomycorrhizal fungi (*L. bicolor* and *H. cylindrosporum*), and two plant-pathogenic fungi (*F. graminearum* and *A. alternata*)*,* all potentially interacting within the rhizosphere of poplars. The present work allows insight into the distinct interactions of *Trichoderma* with ECM or pathogens, and sheds light on the multifaceted responses of *Trichoderma* towards root-associated fungi of different lifestyles, speaking in favor of a clear potential to distinguish between plant’s friend and foes during mycoparasitic confrontations. The described phenomenon of ECM avoidance already at a distance highlights the potential of *Trichoderma* spp. as a promising BCA and bio-fertilizer, while emphasizing the complexities of its interactions with various fungal associates.

## Supplementary Information

Additional file 1.

Additional file 2.

Additional file 3.

Additional file 4.

Additional file 5.

## Data Availability

All data generated or analysed during this study are included in this published article and its supplementary information files. Raw sequencing data is deposited on NCBI SRA server and can be accessed under BioProject number PRJNA1100411 and BioSample numbers SAMN40968214-SAMN40968225.
